# Psychometric properties of the moral injury symptom scale among Chinese health professionals during the COVID-19 pandemic

**DOI:** 10.1186/s12888-020-02954-w

**Published:** 2020-11-25

**Authors:** Wang Zhizhong, Harold G. Koenig, Tong Yan, Wen Jing, Sui Mu, Liu Hongyu, Liu Guangtian

**Affiliations:** 1 Department of Research, Futian Center for Chronic Disease Control, #9 Xinsha Road, Shenzhen, 518000 People’s Republic of China; 2 Department of Epidemiology and Health Statistics, School of Public Health and Management at Ningxia Medical University, Yinchuan, 750004 People’s Republic of China; 3 Department of Medicine, King Abdulaziz University, Jeddah, 21589 Saudi Arabia; 4 Department of Psychiatry, Duke University Medical Center, Durham, NC 27710 USA; 5 Department of Infectious Disease Control, Center for Disease Control and Prevention at Shizuishan City, Shizuishan, 750000 People’s Republic of China; 6 Department of Health Management, School of Public Health and Management, Ningxia Medical University, Yinchuan, 750004 People’s Republic of China; 7 Department of Infectious Disease at the Fourth People Hospital of Ningxia, Yinchuan, 750004 People’s Republic of China

**Keywords:** Moral injury, Moral injury symptom scale, Validation, Reliability

## Abstract

**Background:**

Moral injury among physicians and other health professionals has attracted attention in the mainstream literature, this study aim to assess the psychometric properties of the 10-item Moral Injury Symptoms Scale-Health Professional (MISS-HP) among healthcare professionals in China.

**Methods:**

A total of 583 nurses and 2423 physicians were recruited from across mainland China. An online survey was conducted from March 27 to April 26, 2020 (during the middle of the COVID-19 pandemic) using the Chinese version of the MISS-HP. Reliability was assessed by internal consistency reliability and test-retest reliability. Exploratory factor analysis (EFA) and confirmatory factor analysis (CFA) were performed to determine scale structure.

**Results:**

Cronbach’s α of the scale for both samples was acceptable (0.71 for nurses and 0.70 for physicians), as was test-retest reliability (ICCs for the individual items ranged from 0.41 to 0.74, with 0.77 for the overall scale in physicians). EFA suggested three factors, and the CFA indicated good fit to the data. Convergent validity was demonstrated with the 4-item Expressions of Moral Injury Scale (*r* = 0.45 for physicians, *r* = 0.43 for nurses). Discriminant validity was demonstrated by correlations with burnout and well-being (*r* = 0.34–0.47), and concurrent validity was suggested by correlations with depression and anxiety symptoms (*r* = 0.37–0.45). Known groups validity was indicated by a higher score in those exposed to workplace violence (B = 4.16, 95%CI: 3.21–5.10, *p* < 0.001).

**Conclusions:**

The MISS-HP demonstrated acceptable reliability and validity in a large sample of physicians and nurses in mainland China, supporting its use as a screening measure for moral injury symptoms among increasingly stressed health professionals in this country during the COVID-19 pandemic.

**Supplementary Information:**

The online version contains supplementary material available at 10.1186/s12888-020-02954-w.

## Background

The term moral injury (MI) has increasingly appeared in the research literature since it was first coined by psychiatrist Johnathan Shay in the early 1990s [1]. To date, there are many definitions of MI that have been proposed [2]. More recently, Shay suggested a definition made up of three components: “(1) betrayal of ‘what’s right’ (2) by someone who holds legitimate authority (3) in a high stakes situation” [3]. MI has been found to be present in a wide range of populations experiencing severe trauma, including military personnel, war veterans, first responders, rape victims, and others [4, 5]. At least one qualitative study has reported that the term moral injury is useful for exploring medical students’ experience in emergency medicine settings [6]. A study of refuges in Switzerland found that MI accounted for 16% of the variance in post-tramatic stress disorder (PTSD) symptoms [7]. Papazoglou found that MI was frequently experienced by police officers after suffering repeated trauma [8].

Until 2013, there were no measures to assess MI as currently understood. Since then, several have emerged to assess the presence of MI among military populations, including two types of assessment tools: (1) those that measure both morally injurious events and MI symptoms, and (2) those that measure MI symptoms only. Measures in the first category include the 9-item Moral Injury Events Scale (MIES) developed Nash and colleagues in diverse military samples [9, 10]. Several years later, the 20-item Moral Injury Questionnaire was developed by Currier and colleagues, again assessing both morally-injurious events and symptoms [11]. The first measure to assess MI symptoms only was the 45-item Moral Injury Symptoms Scale-Military Version-Long Form (MISS-M-LF) [12], followed soon by the publication of the 17-item Expressions of Moral Injury Scale-Military Version (EMIS-M) by Currier and colleagues [13]. The MISS-M-LF was then shorted by Koenig and colleagues to a 10-item version (MISS-M-SF) [14], and this was later followed by a 4-item short version of the EMIS-M [15]. Those measures were all developed in samples of active duty military or war veterans.

These scales have largely followed the definitions by Shay [3] and Bret Litz et al. [16] that focused on MI symptoms acquired during combat, such as feelings of shame, grief, meaninglessness, and remorse from having violated core moral beliefs [17]. Symptoms relate to what one has done (killed combatants or innocents, dismembered bodies, maltreated others, or deserted comrades during battle), what one has failed to do (protected innocents or prevented the death of fellow soldiers), and what one has observed others do or fail to do [18]. MI symptoms may also involve intense feelings of betrayal by those in authority, either in or outside of the military, and include religious or spiritual struggles or a complete loss of religious faith resulting from experiences during wartime [17].

Recently, MI among physicians and other health professionals has attracted attention in the mainstream literature, particularly when discussing issues related to burnout [19]. Clinicians may experience MI when they feel their ability to deliver care is compromised by the systems (e.g., insurance, reimbursement, electronic health record) being implemented in hospitals, clinics, and medical practices [20]. During the COVID-19 pandemic, physicians in China have faced difficult ethical/moral decisions given the enormous influx of patients with life-threatening infections and limitations in available ventilators, personal protective equipment, and lifesaving medications. These physicians (and nurses) have had to play God in making decisions on who gets treatment and who does not, as well as having to deal with exposure to the coronavirus themselves and the risk this poses to their families and patients [21, 22]. As a result, health professionals have been stigmatized as vectors of contagion, resulting in their assault, abuse, and isolation during the COVID-19 pandemic, just as they had been during the SARS pandemic [23]. This situation has caused many health professionals to feel a sense of helplessness, shame, and guilt, as hundreds of patients die every day [24]. Unfortunately, until now there have been no psychometrically reliable and valid scales to measure MI symptoms in healthcare professionals.

The purpose of this study is to examine the psychometric properties of the 10-item Moral Injury Symptoms Scale-Health Professional (MISS-HP) developed by Koenig and colleagues [25], which is a modified version of the MISS-M-SF developed in military personnel [13] to make it applicable to healthcare professionals. This measure assesses 10 dimensions of the moral injury: betrayal, guilt, shame, moral concerns, loss of trust, loss of meaning, difficulty forgiving, self-condemnation, religious struggle, and loss of religious/spiritual faith.

## Methods

### Participants and procedure

A convenience sample of physicians and nurses from across mainline China was recruited using a snowball sampling method [26] between May 27 and April 26, 2020. Inclusion criteria were 1) physicians or nurses; and 2) length of practice at least 2 years. The exclusion criteria were: (1) a history of 6 months or more of an extended break from practice for any reason during the past 2 years; (2) inability to use the internet or other mobile devices due to the vision or other disability preventing completion of an online questionnaire; and (3) those not formally licensed to practice medicine or nursing.

**Fig. 1:**
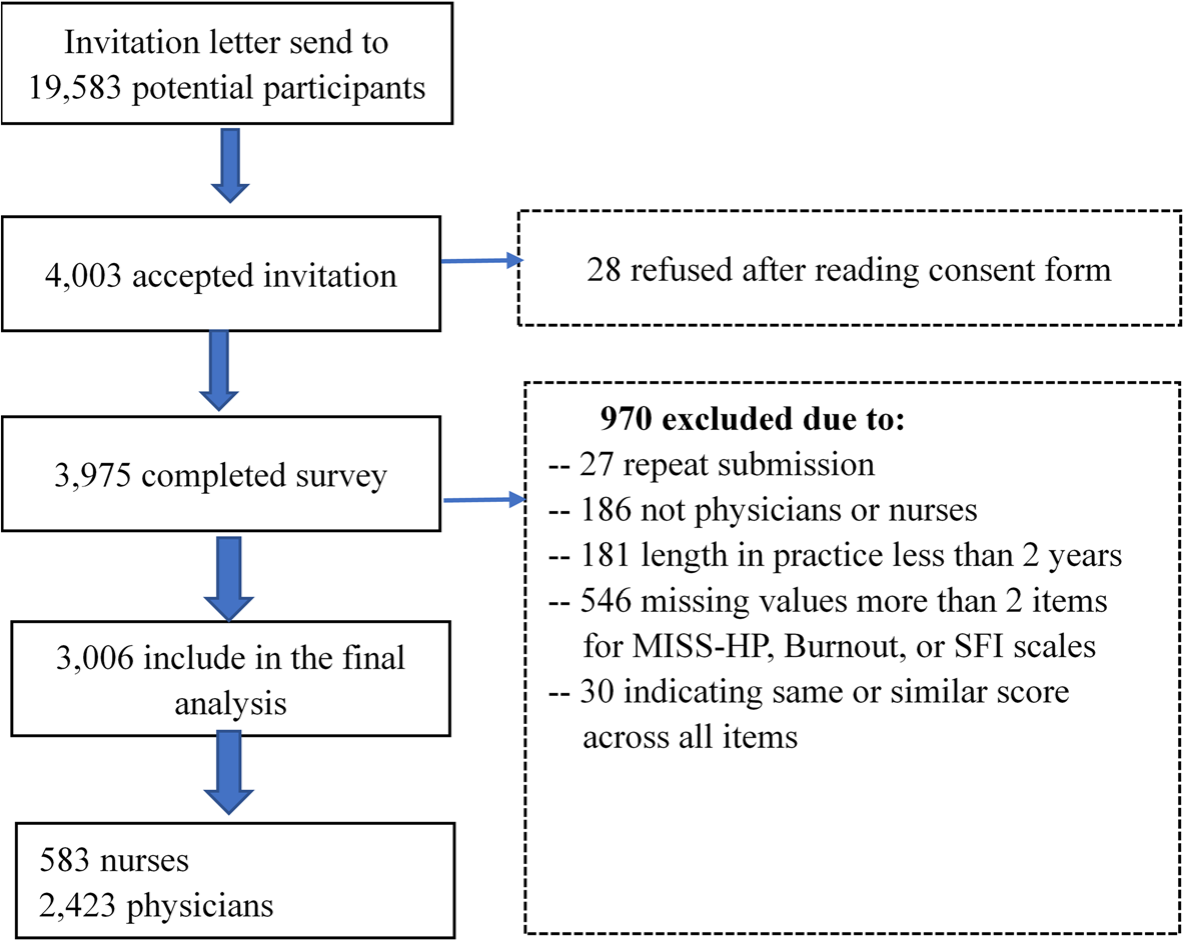
The flowchart of participant enrollment. (MISS-HP: moral injury symptoms scale; SFI: secure flourishing index)

Potential participants were provided a link to an online questionnaire through a popular social media platform (Wechat). Those who responded to the invitation were encouraged to forward the invitation letter to colleagues and post it on social media sites. The invitation letter was initially sent to 19,583 potential participants by the Wechat network, of whom 4003 responded to the invitation; 28 participants refused after reading the informed consent form, resulting in 3975 completed questionnaires (Fig. 1). Of those, 968 records were excluded during the data cleaning process, leaving a final sample of 3006 that consisted of 583 nurses and 2423 physicians who were included in the analysis.

Two-week test-retest reliability was determined by asking 100 physicians from three hospitals to complete the full survey on two occasions, of whom 73 completed the survey at both times.

### Measures

#### Sociodemographic characteristics

Information was collected on age, gender, marital status, educational attainment, ethnicity (Chinese Han vs. minority ethnicity), area of specialty, work area (general medical ward, ICU, emergency room), and length in practice.

#### Moral injury

The Moral Injury Symptom Scale-Health Professional (MISS-HP) is a measure of moral injury symptoms that assesses betrayal, guilt, shame, moral concerns, loss of trust, loss of meaning, difficulty forgiving, self-condemnation, religious struggle, and loss of religious/spiritual faith [25]. Response options for each of the 10 items range from 1 to 10 to signify agreement or disagreement with each statement, with a total score ranging from 10 to 100. The higher scores indicate a greater number and severity of MI symptoms [14].

In order to assess convergent validity, the 4-item Expressions of Moral Injury Scale-Short Form (EMIS-SF) was administered. Developed by Currier and colleagues, this measure has been used widely to assess MI in military personnel [10]. Items were rated on a Likert scale from 1 (strongly disagree) to 5 (strongly agree). Higher total scores indicate the number and severity of MI symptoms, reflecting maladaptive behaviors and internal experiences associated with the moral challenges of delivering clinical care.

#### Mental health

The 9-item Patient Health Questionnaire (PHQ-9) [27] and 7-item Generalized Anxiety Disorder (GAD-7) [28] were used to measure depressive symptoms and anxiety symptoms, respectively. These two instruments are short screening measures frequently used in medical and community settings. Each item on these measures is rated on 4-point Likert scale (from 0 to 3) indicating how often each symptom has occurred within the past 2 weeks. Total scores range from 0 to 54 for PHQ-9 and 0–42 for GAD-7, with higher scores indicating more severe symptoms. The Chinese version of PHQ-9 and GAD-7 scale have strong internal and test-retest reliability as well as strong construct validity and factor structure validity in both medical patients and those in the general population [29, 30].

#### Well-being

The 12-item Secure Flourish Index (SFI) was used to measure six domains of well-being: happiness and life satisfaction, physical and mental health, meaning and purpose, character and virtue, close social relationships, and financial and material stability [31]. Each item was measured on an 11-point visual analogue scale (from 0 to 10), where higher scores indicate higher levels of well-being in each of these areas. Two items assess each of the six domains, and these are averaged to obtain domain-specific scores. The total SFI score is calculated as the average of all six domains with equal weighting. The Chinese version of the SFI has been shown to have acceptable validity and reliability in a Chinese sample [32].

#### Burnout

A modified version of the Maslach Burnout Inventory-Human Services Survey for Medical Personnel (MBI-HSMP) was used to measure the three dimensions of burnout: emotional exhaustion, depersonalization, and reduced personal accomplishment [33]. Each item on the 22-item scale is scored on a 7-point Likert scale from 0 (never) to 6 (daily). Higher scores on each subscale and the overall scale indicate higher levels of burnout. The MBI-HS has been translated into Chinese following a standard procedure, which was shown to have acceptable reliability and validity in a sample composed of participants from a range of occupations [34].

#### Workplace violence

Workplace violence was assessed by asking, “Have you ever been attacked by your patients or their close relatives, either physically or verbally?” Response categories were yes or no.

### Translation of instruments

A 4-step procedure recommended by WHO was used to guide the translation of instruments used in this study into Chinese [35, 36]. First, the original English MISS-HP was translated into Chinese by two health professionals from outside the research team, both of whom were bilingual and fluent in Chinese and English. Next, the two translations were compared and discrepancies reconciled to arrive at a draft Chinese version. Second, a bilingual expert panel consisting of three health professionals (including the original translators) and two social science researchers reviewed the draft Chinese translation separately, making cultural adaptations as necessary. Third, the draft Chinese version was back-translated into English by two bilingual health professionals (different translators than those in the first step). The back-translated English version was then compared to the original English version and reviewed by the original author to ensure that the questions were translated correctly and discrepancies resolved at this stage. Fourth, the draft version of the scale was administrated to 11 physicians from two hospitals for pre-testing. These physicians were asked to send comments about ease of administration, clarity of wording, and time burden. Necessary changes in language were then made based on consensus to arrive at the final Chinese version of the MISS-HP (Supplementary Table 1).

### Data analysis

#### Missing values

When computing scale scores, the mean substitution method was used to replace missing values [37]. If two items or fewer on a scale were missing, we substituted the average score of items answered on the scale for the missing item. If more than two items were missing, the scale score was considered missing and no substitutions were made.

#### Statistical analyses

Descriptive analyses were performed on all subjects depending on whether responses were categorical or continuous. Differences in socio-demographic characteristics between nurses and physicians were tested using the Student’s t-test for continuous variables and the chi-square test for categorical variables. Differences in MISS-HP total scores between different demographic groups were examined using one-way analysis of variance (ANOVA). General linear regression was used to control for covariates.

Convergent/divergent validity was determined by examining correlations between the MISS-HP score and other measures. A correlation matrix was constructed using Pearson correlation coefficients. Cronbach’s alpha was used to assess the internal consistency the of MISS-HP, where alphas equal to or greater than 0.70 are considered acceptable [38]. The intra-class correlation coefficient (ICC) was used to determine 2-week test-retest reliability, where ICCs between 0.41 and 0.60 indicate moderate reliability, those between 0.61 and 0.80 represent good reliability, and those higher than 0.80 indicate excellent reliability [39]. Internal reliability tests were performed separately for the total sample, nurses, and physicians.

Exploratory factor analysis (EFA) and confirmatory factor analysis (CFA) were conducted to extract scale factors. Both physicians and nurses were split randomly into two separate groups. In Group 1 (*n* = 1198 for physicians, *n* = 292 for nurses), EFA was performed using principal components analysis with Promax rotation (an oblique rotation method allowing factors to correlate with each other). The Kaiser-Meyer-Olkin (KMO) index was used to measure sample adequacy, where KMO values of 0.6 or higher indicate adequacy. The Bartlett’s test of sphericity was used to assess the appropriateness of the correlations between variables in the factor model.

In Group 2 (*n* = 1225 for physicians, *n* = 291 for nurses), CFA using the maximum likelihood method was performed to assess the stability of the factor structure. Model adequacy was determined using the chi-square test with degrees of freedom (*df*), where a *p*-value less than 0.05 indicates model adequacy. Indices for model fit included the comparative fit index (CFI), normed fit index (NFI), incremental fit index (IFI), and root mean square error of approximation (RMSEA). The Akaike information criterion (AIC) was also calculated. Values of CFI > 0.90, NFI > 0.90, IFI > 0.90, and RMSEA < 0.08 indicate that model fit is acceptable [40]. All the statistical analyses completed under IBM SPSS 23.0 version software (SPSS Inc., Chicago, IL, USA).

## Results

### Demographic characteristics

Demographic characteristics of the final sample of nurses and physicians are displayed in Table 1. The average age of the overall sample was 35.4 (SD 8.1; range = 20–70 years), and the average length in practice was 11.6 (SD 8.5; range 2–50 years). Approximately one-third of participants were male, and more than half (62.5%) provided inpatient care. Nearly two-thirds (64.2%) of participants reported experiencing workplace violence at some time during their professional practice. Compared with the physicians, nurses were more likely to be female, younger, worked in the ICU or emergency room, had lower educational attainment, and were less likely to experience burnout. Specialty area among physicians was 34% internal medicine, 14% surgery, 12% obstetrics-gynecology or pediatrics, 8% psychiatry, and 31% other specialties.

**Table 1:** Socio-demographic characteristics of participants

	Overall *n* = 3006	Nurses *n* = 583	Physicians *n* = 2423	x^2^/t	*P*
Gender, Male, n (%)	1049 (34.9)	40 (6.9)	1009 (41.6)	250.23	< 0.001
Marital status, n (%)					
Unmarried	656 (21.8)	148 (25.4)	508 (21.0)	6.32	0.042
Married	2266 (75.4)	416 (71.4)	1850 (76.4)		
Divorced/widow	84 (2.8)	19 (3.3)	65 (2.7)		
Ethnic (minorities) n (%)	371 (12.3)	58 (9.9)	313 (12.9)	3.83	0.050
Work area, n (%)					
Inpatient	1878 (62.5)	367 (63.0)	1511 (62.4)	45.06	< 0.001
Outpatient	714 (23.8)	94 (16.1)	620 (25.6)		
ICU/emergency	280 (9.3)	85 (14.6)	195 (8.0)		
Other	134 (4.5)	37 (6.3)	97 (4.0)		
Education, n (%)					
Bachelors degree	2029 (67.5)	568 (97.4)	1461 (60.3)	295.42	< 0.001
Masters	813 (27.0)	14 (2.4)	799 (33.0)		
Ph.D.	164 (5.5)	1 (0.2)	163 (6.7)		
WPV, yes, n (%)	1931 (64.2)	337 (57.8)	1594 (65.8)	13.03	< 0.001
Age, years, M ± SD	35.4 ± 8.1	33.0 ± 7.5	35.9 ± 8.1	62.94	< 0.001
LP, years, M ± SD	11.6 ± 8.5	11.1 ± 8.0	11.7 ± 8.6	2.10	0.147
PHQ-9, M ± SD	10.6 ± 6.0	10.6 ± 6.1	10.6 ± 5.9	0.05	0.815
GAD-7, M ± SD	8.3 ± 5.3	8.1 ± 5.4	8.3 ± 5.2	0.39	0.528
EE, M ± SD	26.0 ± 11.7	23.9 ± 11.8	26.5 ± 11.6	22.45	< 0.001
RPA, M ± SD	30.3 ± 14.1	32.3 ± 10.0	34.2 ± 8.9	18.67	< 0.001
Dep, M ± SD	10.4 ± 6.9	9.3 ± 7.0	10.6 ± 6.8	15.96	< 0.001
SFI, M ± SD	6.3 ± 1.6	6.3 ± 1.6	6.2 ± 1.6	2.53	0.112
MISS-HP, M ± SD	46.9 ± 12.7	46.3 ± 12.2	47.1 ± 12.8	2.11	0.146
EMIS-SF, M ± SD	10.2 ± 3.2	9.9 ± 3.2	10.3 ± 3.2	7.05	0.008

*p < 0.05, **p < 0.01

*M* mean, *SD* standard deviation, *WPV* workplace violence, *LP* length of practice, *MISS-HP* moral injury symptom scale, *EMIS-SF* Expressions of Moral Injury Scale-short form, *PHQ-9* Patient Health Questionnaire, *GAD-7* Generalized Anxiety Disorder, *EE* Emotional Exhaustion, *RPA* Reduced Personal Accomplishment, *Dep* Depersonalization, *SFI* secure flourishing index

### Reliability

As shown in Table 2, The Cronbach’s alpha for the MISS-HP scale when each item was deleted ranged from 0.64 to 0.76 in the overall sample (0.65–0.71 in nurses, 0.63–0.69 in physicians). The Cronbach’s α for the MISS-HP in the overall sample was 0.70 (0.71 in nurses, 0.70 in physicians). Test-retest reliability after 2 weeks indicated ICCs for individual MISS-HP scale items ranging from 0.41 to 0.74; for the total score, the ICC was 0.77. Pearson correlations between the two times of administration were similar to ICCs (results not showed).

**Table 2:** Cronbach’s alpha for the MISS-HP with items removed and total score

Items	Overall (*n* = 3006)		Nurses (*n* = 583)		Physicians (*n* = 2423)		ICC (***n*** = 73)
	M ± SD	α^**a**^	M ± SD	α^**a**^	M ± SD	α^**a**^	
MI1	4.1 ± 2.6	0.70	4.2 ± 2.7	0.69	4.1 ± 2.7	0.69	0.65
MI 2	6.4 ± 3.0	0.64	6.2 ± 3.0	0.66	6.4 ± 3.0	0.64	0.51
MI 3	5.8 ± 3.0	0.64	5.8 ± 3.0	0.65	5.8 ± 3.0	0.63	0.48
MI 4	5.7 ± 3.0	0.64	5.3 ± 2.8	0.66	5.8 ± 3.0	0.64	0.58
MI 5	3.6 ± 2.4	0.70	3.5 ± 2.5	0.71	3.6 ± 2.4	0.69	0.41
MI 6	3.6 ± 2.6	0.70	3.4 ± 2.5	0.71	3.6 ± 2.6	0.69	0.57
MI 7	6.1 ± 2.7	0.70	6.2 ± 2.9	0.70	6.1 ± 2.7	0.69	0.43
MI 8	3.4 ± 2.5	0.69	3.4 ± 2.5	0.70	3.4 ± 2.5	0.68	0.74
MI 9	3.5 ± 2.5	0.66	3.4 ± 2.5	0.68	3.5 ± 2.5	0.66	0.50
MI 10	4.8 ± 2.9	0.69	4.8 ± 3.0	0.69	4.8 ± 2.9	0.69	0.51
**Total**	46.9 ± 12.7	0.70	46.3 ± 12.2	0.71	47.1 ± 12.8	0.70	0.77

**α:** Cronbach’s alpha

^a^Alpha for the individual items refers to alpha for scale if item deleted

*ICC* intraclass correlation coefficients

### Validity

As evidence for convergent validity, a significant positive correlation was found between the MISS-HP and EMIS-SF in both physicians (*r* = 0.45) and nurses (*r* = 0.43) (Table 3). Divergent or discriminant validity was demonstrated by moderate correlations between MISS-HP score and mental health, well-being, and burnout scales. These included PHQ-9 depressive symptoms (*r* = 0.45 for physicians, *r* = 0.37 for nurses), GAD-7 anxiety symptoms (*r* = 0.41 for physicians, *r* = 0.37 for nurses), and similar correlations for the three burnout subscales and well-being measure.

**Table 3:** Correlation matrix for moral injury, mental health, burnout, and well-being

	1	2	3	4	5	6	7	8
1.MISS	1	0.45^**^	0.45^**^	0.41^**^	0.42^**^	−0.28^**^	0.42^**^	−0.50^**^
2.EMIS	0.43^**^	1	0.47^**^	0.46^**^	0.36^**^	−0.10^**^	0.37^**^	−0.33^**^
3.PHQ	0.37^**^	0.47^**^	1	0.81^**^	0.62^**^	−0.20^**^	0.53^**^	−0.61^**^
4.GAD	0.37^**^	0.53^**^	0.77^**^	1	0.60^**^	− 0.18^**^	0.49^**^	−0.55^**^
5. EE	0.34^**^	0.33^**^	0.62^**^	0.62^**^	1	−0.06^**^	0.74^**^	−0.53^**^
6. RPA	−029^**^	−0.12^**^	− 0.09^*^	−0.09^*^	0.02	1	−0.22^**^	0.39^**^
7. Dep	0.40^**^	0.38^**^	0.59^**^	0.57^**^	0.78^**^	−0.14^**^	1	−0.52^**^
8. SFI	−0.47^**^	−0.37^**^	− 0.54^**^	−0.53^**^	− 0.49^**^	0.39^**^	− 0.54^**^	1

In bold is the correlation matrix for physicians (*n* = 2423); left part is the correlation matrix for nurses (*n* = 583)

^*^p < 0.05, ^**^p < 0.01

*M* mean, *SD* standard deviation, *MII* moral injury index, *EMIS* Expressions of Moral Injury Scale, *PHQ* Patient Health Questionnaire, *GAD* Generalized Anxiety Disorder, *EE* Emotional Exhaustion, *RPA* Reduced Personal Accomplishment, *Dep* Depersonalization, *SFI* secure flourishing index

Known groups validity was supported by comparing MISS-HP scores between those who reported workplace violence and those who did not. As indicated in Table 4, health professionals who experienced workplace violence scored higher on the MISS-HP and EMIS-SF score than those who did not (*p* < 0.01). After controlling demographic variables, workplace violence was significantly correlated with MI symptoms (*B* = 4.16, 95% CI = 3.21–5.10, *p* < 0.001).

**Table 4:** Moral injury score and workplace violence exposure

	Nurses (*n* = 583)		Physicians (*n* = 2423)	
	no	yes	no	yes
Moral Injury Symptoms Scale				
M ± SD	44.2 ± 12.2	47.8 ± 11.9	44.8 ± 12.6	48.4 ± 12.7
*t / P*	12.21 / 0.001		44.29 / < 0.001	
Expressions of Moral Injury Scale				
M ± SD	9.4 ± 3.3	10.3 ± 3.0	9.8 ± 3.2	10.5 ± 3.1
*t / P*	10.72 / 0.001		28.10 / < 0.001	

*M* mean, *SD* standard deviation

Construct validity was examined by exploratory factor analysis (EFA) followed by confirmatory factor analysis (CFA). EFA in the nurses’ sample (Group 1) revealed a KMO index = 0.72, and the Bartlett’s test of sphericity indicated the sample was factorable at *p* < 0.001 (*X*^*2*^_45_ = 6.49E^2^). As illustrated in Supplementary Figure 1, the three extracted factors explained 59.2% of the total variance. The EFA in the physicians’ sample (Group 1) revealed a KMO index of 0.73, and the Bartlett’s test of sphericity demonstrateed factorability at *p* < 0.001 (*X*^*2*^_45_ = 5.27E^3^). As in nurses, three factors were extracted that explained 58.9% of the total variance. As indicated in Table 5, factor 1 (“shame and guilty”) included items MI2, MI3, and MI4, whereas factor 2 (“mistrust”) included items MI5, MI6, and MI10, and factor 3 (“forgiveness”) made up of four items MI1, MI7, MI8, and MI9.

**Table 5:** The factor structure model of the MISS-HF

Items	Nurses (583)			Physicians (*n* = 1198)		
	Factor Component			Factor Component		
	1	2	3	1	2	3
MI1	0.45	0.24	**0.40**	0.15	0.09	**0.61**
MI 2	**0.84**	−0.12	0.03	**0.83**	−0.11	0.08
MI 3	**0.79**	−0.19	0.13	**0.84**	−0.11	0.14
MI 4	**0.74**	−0.07	0.19	**0.69**	−0.05	0.30
MI 5	−0.09	**0.76**	0.16	−0.17	**0.71**	0.15
MI 6	−0.10	**0.78**	0.19	−0.09	**0.80**	0.15
MI 7	−0.01	0.37	**0.54**	0.19	0.44	**0.62**
MI 8	0.15	0.18	**0.74**	0.26	0.30	**0.61**
MI 9	0.20	0.08	**0.75**	0.30	0.09	**0.66**
MI 10	−0.08	**0.70**	−0.17	−0.03	**0.74**	−0.05

Items in the factor are marked in bold

**Fig. 2:**
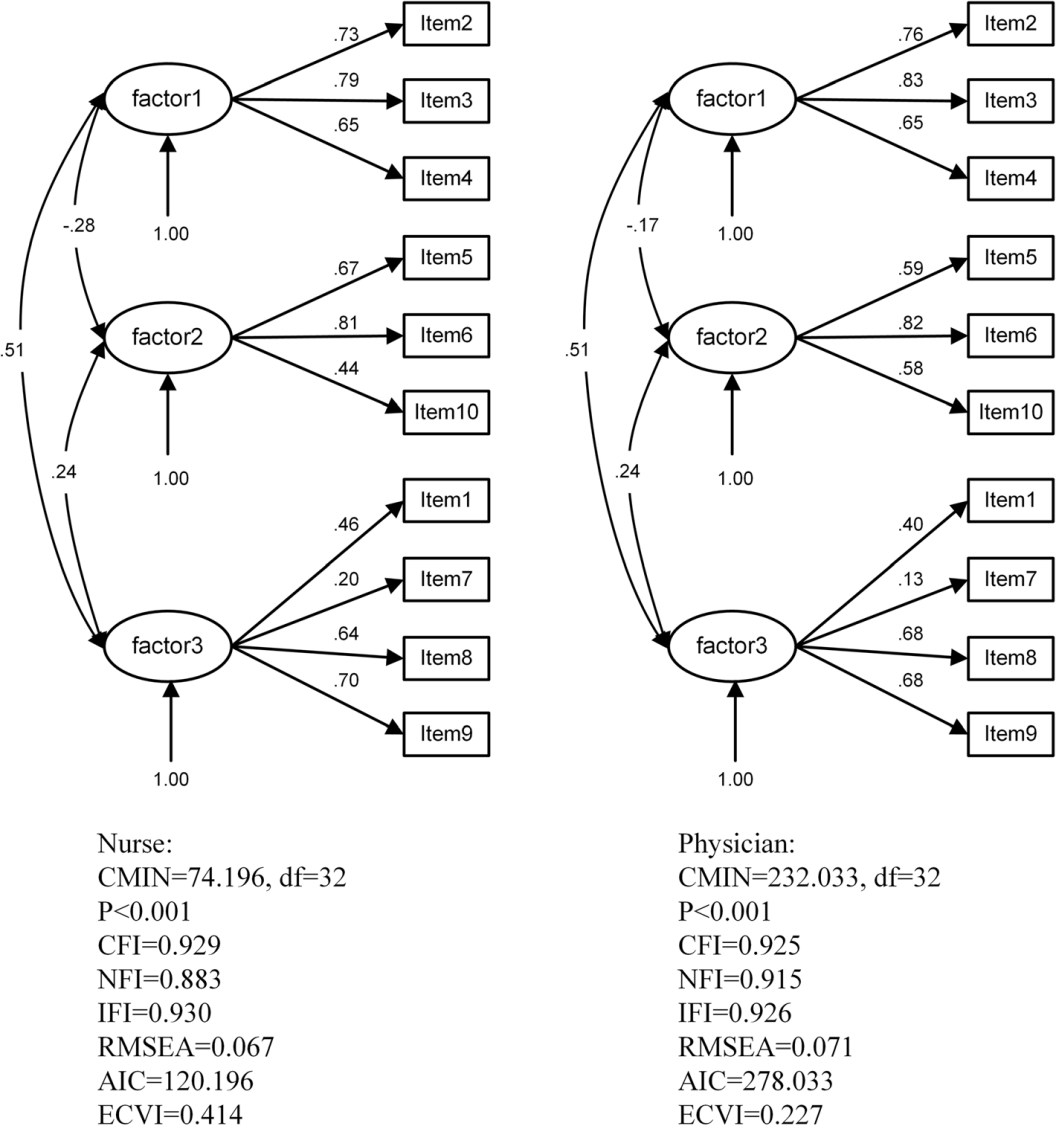
The confirmatory factor analysis models

CFA confirmed the three factor model for the MISS-HP scale in nurses (χ2 = 74.19; df = 32; *p* < 0.001, CFI = 0.93, NFI = 0.88, IFI = 0.93, RMSEA = 0.067, AIC =120.19, and ECVI =0.414). Likewise, CFA confirmed the three factor model in physicians (χ2 = 232.03; df = 32; *p* < 0.001, CFI = 0.93, NFI = 0.92, IFI = 0.93, RMSEA = 0.071, AIC = 278.03, and ECVI =0.23) (see Fig. 2).

## Discussion

To our knowledge, this is the first study to examine the psychometric properties of the MISS-HP, a short but comprehensive measure of moral injury symptoms, in a large sample of health professionals. Unlike other measures of MI, the MISS-HP is unique in that it assesses both psychological and religious/spiritual dimensions of MI. The results indicated that the MISS-HP is a reliable and valid measure of MI in both nurses and physicians. The findings provide primary evidence supporting the use of this tool for assessing symptoms of MI as part of health promotion programs for health professionals in China. The MISS-HP also fills an important gap in research that examines the prevalence, correlates, and health consequences of MI in nurses and physicians.

The internal consistency of the MISS-HP (alpha = 0.70 for physicians and 0.71 for nurses) is acceptable, as is the test-retest reliability (ICC = 0.77 in physicians). With regard to validity, the MISS-HP has acceptable convergent validity with another established measure of MI, the EMIS-SF (*r* = 0.45 for physicians and *r* = 0.43 for nurses). Correlations with common mental conditions (depression and anxiety), well-being, and burnout measures are as robust with the MISS-HP as with the EMIS-SF.

Known groups validity supports using the MISS-HP to identify MI among those suffering from potentially morally injurious events such as being assaulted by patients or relatives. This finding is partly supported by a study of military veteran family members, which found that such violence inflicts damage to moral belief systems and causes a loss of trus t [41]. Many physicians have been killed and injured during the past decade in China [42]. Moral injury can be the consequence of unexpected violence from patients or their relatives, giving rise to feelings of betrayal in nurses and physicians by the very population they are risking their lives to help (especially during this COVID-19 pandemic) [16].

Construct validity of the MISS-HP was established using exploratory factor analysis (EFA), which was then verified by CFA. Factor analysis indicated a three-dimensional structure for the MISS-HP, explaining 59% of the total variance. This finding is consistent with the work of Griffin and colleagues [2] who suggested at least two interrelated MI symptom dimensions, self-directed outcomes (e.g., thoughts/feelings of responsibility for occurrence of moral violations such as shame or viewing oneself as unlovable or unforgivable) and other-directed outcomes (e.g., thoughts/feelings associated with being a victim of others’ morally transgressive acts). Add to this the religious/spiritual dimension of MI involving struggle and loss of faith.

### Limitations

Several aspects of the present study limit the generalizability of these findings, thereby influencing both research and clinical implications. First, we assessed the MISS-HP in a single cross-sectional study involving a nonrandom sample of Chinese health professionals which did not include those in practice for less than 2 years (who may be at even greater risk if MI given their lack of experience). The the findings here require cautious generalization to service members in other areas of the China and to health professionals outside of China. Second, although, a standard translation procedure was used to create a Chinese version of the MISS-HP, cultural differences between China and the Western society (where the scale as initially developed and designed) may have affected the final result (both the translation and the meaning of items). Third, despite the consistent findings showed in nurses and physicians, test-retest relieability was conducted only in physicians, which may lead to uncertainty for the scale’s use in nurses. Fourth, the internal reliability of the MISS-HP was borderline but acceptable in both nurses and physicians (alpha = 0.70 or higher). Fifth, other morally injurious events besides workplace violence need to be assessed in future studies. Finally, like all self-report measures, the accuracy of responses cannot be guaranteed where external factors may influence the report of symptoms (even though the survey was anonymous in nature).

## Conclusions

The 10-item MISS-HP is a brief, comprehensive, reliable, and valid measure for assessing symptoms of moral injury in physicians and nurses providing healthcare to patients in mainland China during the COVID-19 pandemic. Scores on the scale of 50 or higher have been found to indicate significant difficulty with social and occupational functioning in this population [41]. From both a clinical and research perspective, the MISS-HP can be used to screening for MI symptoms and follow response to treatment among healthcare professionals in China.

## Supplementary Information

**Additional file 1: Supplementary Table 1.** The Chinese Version of Moral Injury Symptom Scale with Original English Version. **Supplementary Figure 1.** Scree plot of eigenvalues.

## Data Availability

Data in request to Wang ZZ at wzhzh_lion@126.com. This paper does not include any information about patients with COVID-19, and the data reported in this paper has not included in any other reports.

## Abbreviations

MI: Moral injury
PTSD: Posttramatic stress disorder
MIES: Moral injury events scale
MISS-M-LF: Moral Injury Symptoms Scale-Military Long Form
EMIS-M: Expressions of Moral Injury Scale-Military version
MISS-HP: Moral Injury Symptoms Scale-Health Professional
MISS-M-SF: Moral Injury Symptoms Scale-Military Short Form
PHQ-9: Patient Health Questionnaire
GAD-7: Generalized Anxiety Disorder
SFI: Secure Flourish Index
MBI-HSMP: Maslach Burnout Inventory-Human Services Survey for Medical Personnel
ANOVA: One-way analysis of variance
ICC: Intra-class correlation coefficient
EFA: Exploratory factor analysis
CFA: Confirmatory factor analysis
KMOss: Kaiser-Meyer-Olkin
CFI: Comparative fit index
NFI: Normed fit index
IFI: Incremental fit index
RMSEA: Root mean square error of approximation
AIC: Akaike information criterion
SD: Standard deviation

## References

[1] Shay J, Munroe J, Saigh PA, Bremner JD (1998). Group and milieu therapy for veterans with complex posttraumatic stress disorder. Posttraumatic stress disorder: a comprehensive text.

[2] Griffin BJ, Purcell N, Burkman K, Litz BT, Bryan CJ, Schmitz M, Maguen S (2019). Moral injury: an integrative review. J Trauma Stress.

[3] Shay J (2014). Moral injury. Psychoanal Psychol.

[4] Bryan AO, Bryan CJ, Morrow CE, Etienne N, Ray-Sannerud B (2014). Moral injury, suicidal ideation, and suicide attempts in a military sample. Traumatology.

[5] Battles AR, Bravo AJ, Kelley ML, Whit TD, Braitman AL, Hamrick HC (2018). Moral injury and PTSD as mediators of the associations between morally injurious experiences and mental health and substance use. Traumatology.

[6] Murray E, Krahé C, Goodsman D (2018). Are medical students in prehospital care at risk of moral injury?. Emerg Med J.

[7] Nickerson A, Schnyder U, Bryant RA, Schick M, Mueller J, Morina N (2015). Moral injury in traumatized refugees. Psychother Psychosom.

[8] Papazoglou K, Chopko B. The role of moral suffering (moral distress and moral injury) in police compassion fatigue and PTSD: an unexplored topic. Front Psychol. 2017;8:1999. 10.3389/fpsyg.2017.01999. 10.3389/fpsyg.2017.01999 PMC5694767 29187830

[9] Nash WP, Marino Carper TL, Mills MA, Au T, Goldsmith A, Litz BT (2013). Psychometric evaluation of the moral injury events scale. Mil Med.

[10] Bryan CJ, Bryan AO, Anestis MD, Anestis JC, Green BA, Etienne N (2016). Measuring moral injury: Psychometric properties of the moral injury events scale in two military sample. Assessment.

[11] Currier JM, Holland JM, Drescher K, Foy D (2015). Initial psychometric evaluation of the moral injury questionnaire -military version. Clin Psychol Psychother.

[12] Koenig HG, Ames D, Youssef NA, Oliver JP, Volk F, Teng EJ, Pearce M (2018). The moral injury symptom scale-military version. J Relig Health.

[13] Currier JM, Farnsworth JK, Drescher KD, McDermott RC, Sims BM, Albright DL (2018). Development and evaluation of the expressions of moral injury scale—military version. Clin Psychol Psychother.

[14] Koenig HG, Ames D, Youssef NA, Oliver JP, Volk F, Teng EJ, Pearce M (2018). Screening for moral injury: the moral injury symptom scale–military version short form. Mil Med.

[15] Currier JM, Isaak SL, McDermott RC (2019). Validation of the Expressions of Moral Injury Scale-Military version-Short Form. Clin Psychol Psychother.

[16] Litz BT, Stein N, Delaney E, Lebowitz L, Nash WP, Silva C, Maguen S (2009). Moral injury and moral repair in war veterans: a preliminary model and intervention strategy. Clin Psychol Rev.

[17] Brock RN, Lettini G. Soul repair: recovering from moral injury after war. Boston: Beacon Press; 2012. USA, P xiv.

[18] Koenig HG, Youssef NA, Pearce M (2019). Assessment of Moral Injury in Veterans and Active Duty Military Personnel With PTSD: A Review. Front Psychiatry.

[19] Kopacz MS, Ames D, Koenig HG (2019). It's time to talk about physician burnout and moral injury. Lancet Psychiatry.

[20] Ford EW (2019). Stress, burnout, and moral injury. J Healthc Manag.

[21] Lu W, Wang H, Lin Y, Li L. Psychological status of medical workforce during the COVID-19 pandemic: a cross-sectional study. Psychiatry Res. 2020;288:e112936. doi:10.1016/j.psychres.2020.112936. 10.1016/j.psychres.2020.112936 PMC7195354 32276196

[22] Fava GA, McEwen BS, Guidi J, Gostoli S, OffidaniE SN (2019). Clinical characterization of allostatic overload. Psychoneuroendocrinology.

[23] Person B, Sy F, Holton K, Govert B, Liang A (2004). Fear and stigma: the epidemic within the SARS outbreak. Emerg Infect Dis.

[24] BBC news. Coronavirus: Why healthcare workers are at risk of moral injury. https://www.bbc.com/news/world-us-canada-52144859. Accessed May 5th, 2020.

[25] Mantri S, Koenig HG, Wang ZZ, Lawson J. Identifying Moral Injury in Healthcare Professionals: The Moral Injury Symptoms Scale-HP. J Relig Health. 2020, online ahead. 10.1007/s10943-020-01065-w. 10.1007/s10943-020-01065-w PMC7366883 32681398

[26] Baltar F, Brunet I (2012). Social research 2.0: virtual snowball sampling method using Facebook. Internet Res.

[27] Kroenke K, Spitzer RL, Williams JB (2001). The PHQ-9: validity of a brief depression severity measure. J Gen Intern Med.

[28] Spitzer RL, Kroenke K, Williams JB, Lowe B (2006). A brief measure for assessing generalized anxiety disorder: the GAD-7. Arch Intern Med.

[29] Zhang YL, Liang W, Chen ZM (2013). Validity and reliability of patient health Questionnaire-9 and patient health Questionnaire-2 to screen for depression among college students in China. Asia Pac Psychiatry.

[30] He XY, Li CB, Qian J, Cui HS, Wu WY. Reliability and validity of a generalized anxiety scale in general hospital outpatients. Shanghai Arch Psychiatry. 2010;22(4):200–3.

[31] VanderWeele TJ (2017). On the promotion of human flourishing. Proc Natl Acad Sci U S A.

[32] Wȩziak-Białowolska D, McNeely E, VanderWeele TJ (2019). Human Flourishing in Cross Cultural Settings. Evidence From the United States, China, Sri Lanka, Cambodia, and Mexico. Front Psychol.

[33] Maslach C, Jackson SE, Leiter MP (1996). Maslach burnout inventory manual.

[34] Wang Y, Zhang H, Lei J, Yu Y (2019). Burnout in Chinese social work: differential predictability of the components of the Maslach burnout inventory. Int J Soc Welf.

[35] World Health Organization. Process of translation and adaptation of instruments. Access on January 6th, 2020. https://www.who.int/substance_abuse/research_tools/translation/en/.

[36] Gudmundsson E (2009). Guidelines for translating and adapting psychological instruments. Nordic Psychology.

[37] Downey RG, King CV (1998). Missing data in Likert ratings: a comparison of replacement methods. J Gen Psychol.

[38] Bland JM, Altman DG (1997). Cronbach's alpha. BMJ (Clinical Res ed).

[39] Bartko JJ (1966). The intraclass correlation coefficient as a measure of reliability. Psychol Rep.

[40] Bentler PM (1999). Cutoff criteria for fit indexes in covariance structure analysis: conventional criteria versus new alternatives. Struct Equ Model.

[41] Pan Y, Hong Yang X, He JP (2015). To be or not to be a doctor, that is the question: A review of serious incidents of violence against doctors in China from 2003–2013. J Public Health.

[42] Wang ZZ, Koenig HG, Tong Y, Wen J, Sui M, Liu H, Al Zaben F, Liu G. Moral injury in Chinese health professionals during COVID-19 pandemic. Br J Psychiatry. 2020;2020 in submission. 10.2139/ssrn.3606455.

