# Supplementary material for: Psychometric properties of the moral injury symptom scale among Chinese health professionals during the COVID-19 pandemic
Source: BMC Psychiatry. 2020 Nov 25;20:556. doi: 10.1186/s12888-020-02954-w (PMC7686837; doi:10.1186/s12888-020-02954-w)
Supplement: Supplementary file 1 — Additional file 1: Supplementary Table 1. The Chinese Version of Moral Injury Symptom Scale with Original English Version. Supplementary Figure 1. Scree plot of eigenvalues. [file 12888_2020_2954_MOESM1_ESM.docx]

**Supplementary Table 1** The Chinese Version of Moral Injury Symptom Scale with Original English Version

| **Final Chinese version** | **Original English scale** |
| --- | --- |
| **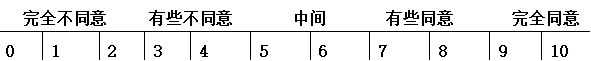** | **Strongly Mildly Neutral Mildly Strongly**  **disagree disagree agree agree**  **1 2 3 4 5 6 7 8 9 10** |
| 我觉得那些曾经信任的同事辜负了我 | I feel betrayed by other health professionals whom I once trusted. |
| 我因没能成功挽救危重病人生命而感到内疚 | I feel guilt over failing to save someone from being seriously injured or dying |
| 我因为在工作过程中没能给予患者应该的诊疗服务而觉得惭愧 | I feel ashamed about what I’ve done or not done when providing care to my patients |
| 我因为不能依照自己的道德标准和价值观开展诊疗活动而烦恼 | I am troubled by having acted in ways that violated my own morals or values |
| 我在工作中遇到的大多数人都是值得信赖的 | Most people with whom I work as a health professional are trustworthy |
| 作为一名医务人员，我感到自己的人生非常有意义 | I have a good sense of what makes my life meaningful as a health professional |
| 我已经原谅了我的失误和对患者造成的伤害 | I have forgiven myself for what’s happened to me or to others whom I have cared for |
| 总而言之，我觉得自己是一位失败的医务人员 | All in all, I am inclined to feel that I’m a failure in my work as a health professional |
| 有时我觉得，是因为自己工作中的失误而被老天爷惩罚 | I sometimes feel God is punishing me for what I’ve done or not done while caring for patients |
| 在经历了上述问题之后，我的宗教信仰（精神信念）更加坚定了 | Compared to before I went through these experiences, my religious/spiritual faith has strengthened |


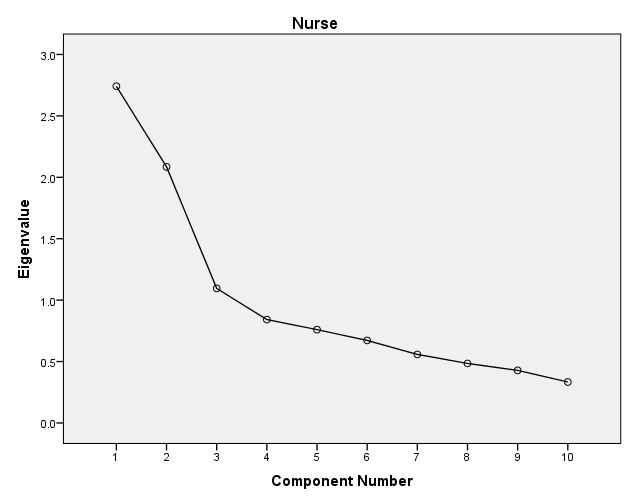


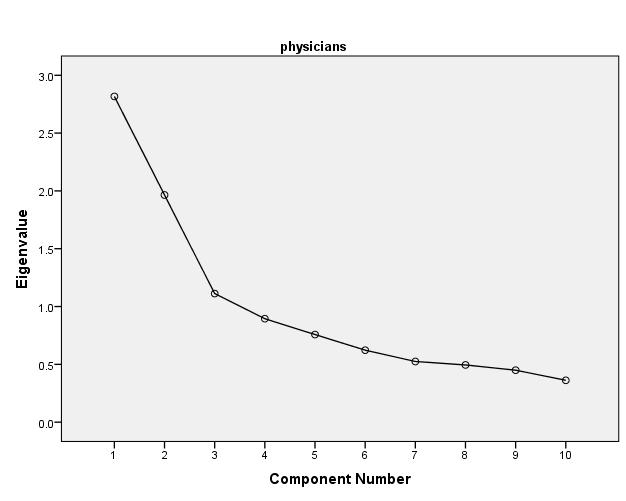


**Supplementary Figure 1.** Scree plot of eigenvalues
